# How traumatic internship experiences shape midwifery students' motivation and career expectations: Findings from a qualitative study

**DOI:** 10.18332/ejm/205672

**Published:** 2025-07-03

**Authors:** Martina Dato, Mayra Veronese, Renzo Zanotti, Matteo Danielis

**Affiliations:** 1 Laboratory of Studies and Evidence Based Nursing, Department of Cardiac, Thoracic, Vascular Sciences and Public Health, University of Padua, Padua, Italy

**Keywords:** traumatic event, student, motivation, internship, phenomenological study

## Abstract

**INTRODUCTION:**

Traumatic events experienced during midwifery internships can have a significant impact on students, leading to emotional distress and doubts about continuing their studies. The aim of this study was to explore whether the experience of traumatic events influences motivation to continue studying and affects the professional expectations associated with the role of the midwife.

**METHODS:**

A qualitative descriptive phenomenological approach was used, focusing on the lived experiences of midwifery students in Italy. Data were collected through open-ended questionnaires administered to 36 third-year students from a midwifery Bachelor's degree program during the 2023-2024 academic year. Thematic analysis, following the Braun and Clarke six-step framework, was used to identify and interpret patterns in the data.

**RESULTS:**

The analysis revealed 21 codes, eight subthemes, and three main themes: ‘Discovering the elements that shape the perception of the event’, ‘Suffer the consequences of the event’, and ‘Engage in a dynamic process to acquire the professional dimension’. Students described the factors that shape their perception of events, such as inadequate preparation, emergencies, and negative professional behaviors. Emotional consequences included fear, frustration, and doubts about their professional abilities. Despite these challenges, students showed resilience, often seeking support from mentors and external resources, ultimately reaffirming their commitment to the profession of midwifery.

**CONCLUSIONS:**

Traumatic events during internships significantly impact the experiences of midwifery students. Effective clinical tutoring and emotional support are crucial to mitigate negative effects and foster professional growth. Understanding these dynamics can help educators better prepare students for the emotional demands of the profession.

## INTRODUCTION

The internship represents a key component of the learning process for midwifery students, offering them the opportunity to apply theoretical knowledge in a clinical setting. However, it also constitutes one of the most demanding periods of their education process^1^. During this period, students may face common stressors, such as adapting to a new setting or assuming a new professional role. Furthermore, they may be exposed to traumatic events which, due to their vulnerability, can be perceived as particularly stressful or even traumatic^2-4^. Traumatic events represent any disturbing experience that causes significant fear, confusion, or other intensely disruptive feelings that have a lasting negative effect on the attitudes and behavior of a person^5^. In the clinical setting, traumatic events may include patient death, cases of severe neglect or abuse, violent events, or emergencies^2,6,7^.

Although not all students experience traumatic events during their internship, those who do experience them are often unprepared and may lack the coping mechanisms required to effectively manage their impact, which can lead to long-term psychological effects such as post-traumatic stress disorder, burnout, or psychological trauma^7-9^. For midwifery students, clinical internships, particularly in the delivery room, are physically and emotionally demanding, as they require managing complex processes such as traumatic deliveries, stillbirths, postpartum hemorrhages, and neonatal resuscitations, all of which may contribute to the development of fear related to childbirth^10,11^. Furthermore, previous studies have highlighted that workplace bullying, often present in high-pressure clinical settings, can exacerbate students’ perception of traumatic events and undermine their ability to learn effectively and develop a secure professional identity^12,13^. Edmonson and Zelonka^13^ emphasize that nurse bullying is a pervasive problem that begins before nursing school and continues throughout a nurse’s career, contributing to poor work environments, increased patient risk, and significant turnover costs for healthcare institutions.

Experience of traumatic events, combined with the intense empathetic relationship established with women, can increase the vulnerability of students to psychological distress^11^ and has also been linked to a tendency among midwives to adopt defensive practice or depersonalize those in their care, with significant implications for women’s experiences throughout the perinatal period^14^. The stress that arises from these episodes, along with inadequate preparation to handle such situations, often causes motivation loss, leads to burnout, reduces compassionate feelings during the care process, and affects students’ ability to continue their studies^10,15,16^. Davies and Coldridge^15^ conducted a qualitative study in Northwest England to explore midwifery students’ experiences of traumatic events during internships. The findings revealed that students often felt overwhelmed by responsibilities, perceived themselves as lacking the resilience to cope, and, in some cases, questioned continuing their studies, whether due to the direct impact of traumatic events or a broader sense of disillusionment with the profession^15^.

Given the significant impact that traumatic events can have on midwifery students, it is crucial to conduct more in-depth investigations into this phenomenon in diverse academic contexts. This research is necessary to develop a comprehensive understanding of the consequences of these experiences in the Italian context. This study aims to examine how the experience of traumatic events during clinical internships influences the motivation of students to continue their studies and shapes their expectations of the professional role of the midwife.

## METHODS

### Study design

A qualitative descriptive phenomenological approach was adopted. This approach focusses on lived experiences and aims to gain a deep understanding of how people perceive and experience the world^17^. Rather than interpreting the phenomenon, it aims to describe it in its unique details. Methods and findings are reported by Qualitative research review (RATS) guidelines^18^.

### Participants

A purpose sampling strategy was used for the study^19^. All third-year students (a total of 40 students) enrolled in the Bachelor’s degree in midwifery at Padua University during the 2023–2024 academic year were invited to participate. Third-year students were selected for the study because they had completed the majority of their clinical internships and were approaching the crucial transition from student to professional midwife. According to the International Confederation of Midwife Global Standards for Midwifery Education, midwifery students are required to balance theoretical studies with clinical practice, dedicating a large part of their curriculum (a minimum of 50%) to clinical practice^20^. In Italy, the Bachelor’s degree in midwifery lasts three years. During the program, midwifery students complete an internship of approximately 1800 hours of supervised clinical practice. In the Veneto region, students completed their internships across five hospitals. Three of these were referral centers, each handling between 2300 and 2900 births annually. The other two hospitals had lower birth rates, ranging from approximately 500 to 1000 deliveries per year.

### Data collection

To explore the phenomenon, an open-ended survey was chosen (Supplementary file Table 1), consisting of two parts: 1) Sociodemographic information about the students, including their age, gender, whether they exceeded the standard time to graduate, and the main reason for enrolling in the Bachelor’s degree in midwifery; and 2) Open-ended questions designed to explore in depth the experience of the traumatic event, asking participants to describe what happened, who was involved, the behaviors that contributed to making the event traumatic, the emotions experienced, and the consequences the event had on them.

Data were collected from 5 to 18 June 2024. The midwifery educator of the Bachelor’s degree in midwifery sent formal invitations to students via email. To accommodate students’ diverse schedules and maximize participation, three different interview sessions were arranged. This approach allowed students to choose the most convenient time slot, thereby increasing the likelihood of their involvement in the study. Data were collected by the first author, a Master’s degree student in Nursing and Midwifery Sciences and a registered midwife five years experienced, supervised by a senior midwife with previous experience in education and research. The first author met the students who volunteered to participate in the study and administered the questionnaire in a university classroom where no faculty members from the Bachelor’s degree program were present. The midwifery students were given 60 minutes to complete the questionnaire. To ensure anonymity regarding the hospital where they attended their internship, participants were instructed to use fictitious names when describing events in their responses. Once completed, the questionnaires were collected in a single envelope and mixed. The questionnaires were reviewed only after the entire data collection period had ended.

### Data analysis

The questionnaires were transcribed verbatim and subsequently uploaded into Atlas.ti^®^ version 24 for inductive thematic analysis, which followed the six steps outlined by Braun and Clarke^21^. The first step involves familiarizing with the data, often requiring repeated reading to go deeper into the content. The second step consists of generating the initial codes, where researchers assign a ‘label’ to a sentence or short passage of text that they consider to be particularly representative of specific topic. The third step focused on searching for themes. During this phase, the codes were grouped into potential themes and considering the relationship between codes and themes, as well as between different levels of themes (e.g. subthemes and main themes). In the fourth step, the themes were reviewed and refined. This process included creating a thematic map to visualize the relationships among themes and to ensure that they were coherent, distinct and representative of the data. The fifth step consisted of defining and naming the themes. This involved clearly articulating the essence of each theme and ensuring that the subthemes were appropriately aligned with their main themes. Finally, in the sixth step, the findings were synthesized into a detailed report. This report encapsulated the key themes, subthemes, and their relationships, illustrating the experiences and insights of the students^21^.

The coding process was initially developed by two authors (MD and MV), then refined with the involvement of the entire research. To ensure accuracy and reliability, all authors discussed and refined the codes. All disagreements were resolved by discussion.

### Ethics

Permission to conduct the study was granted by the Academic Board of the Padua University, which officially approval communicated on 23 May 2024. Before data collection, the principal investigator provided participants with detailed information about the study’s purpose and addressed any questions they had. Written informed consent was obtained from all participants prior to data collection. The data collected were completely anonymous and treated according to General Data Protection Regulation (UE 2016/679) and the Declaration of Helsinki^22^. To protect student anonymity, each quote was assigned a unique code (e.g. S1 = Student 1).

## RESULTS

A total of 36 third-year students from the Midwifery Bachelor’s degree program participated in the study (response rate of 90%). Of the original cohort, two students were abroad at the time of data collection, and two others did not provide their consent to participate. As shown in Table 1, the entire sample was female, with an average age of 23.3 years, ranging from 21 and 41 years. Almost all the students completed the program within the standard time frame for graduation.

**Table 1:** Sociodemographic characteristics of participants (N=36)

*Characteristics*	*n (%)*
**Gender**	
Female	36 (100)
**Age** (years)	
Mean (SD)	23.3 (3.3)
Median (range)	23 (21–41)
**Has the student exceeded the standard time to graduate?**	
No	35 (97.2)
Yes	1 (2.8)

**Figure 1:**
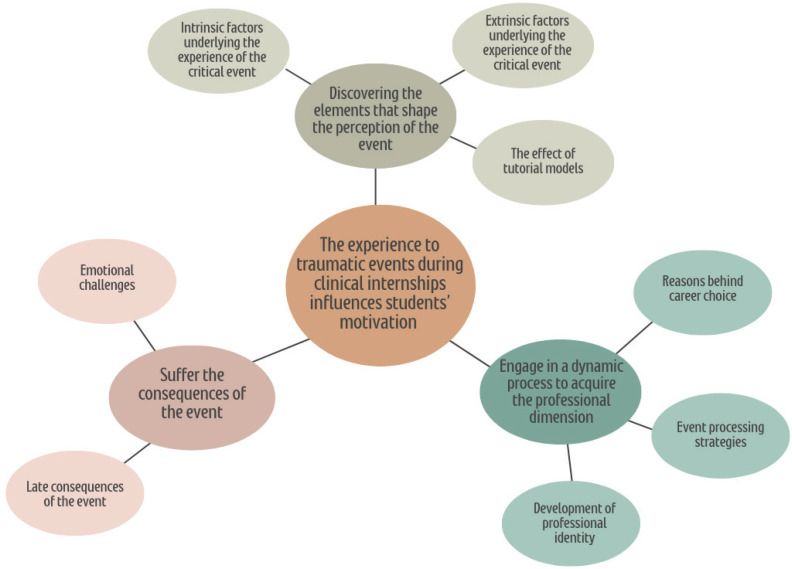
Coding tree

We identified twenty-one codes, organized into eight subthemes, and three themes (Figure 1). Finally, three main themes emerged from the data (Table 2): 1) ‘Discovering the elements that shape the perception of the event’, 2) ‘Suffer the consequences of the event’, and 3) ‘Engage in a dynamic process to acquire the professional dimension’.

**Table 2:** Description of the coding tree

*Themes*	*Subthemes*	*Codes*	*Quotations*
**Theme 1: Discovering the elements that shape the perception of the event**	Intrinsic factors underlying the experience of the critical event	Poor understanding of what is happening	*‘I don’t understand anything anymore; I just feel a lot of agitation, the woman screaming, and my mentor asking me to fetch things that I don’t know where to find.’* (S29)
		Lack of experience	*‘I realized that it takes experience to become impassive during emergencies, and that it would never have been possible for me to be more composed during my first experience: I forgive myself, even if perhaps my clinical preceptor at the time did not understand that I only needed to gain experience.’* (S18)
		Awareness of one’s own limitations	*‘Even though I was still familiarizing with the specific topics of childbirth, I was regularly inappropriately reprimanded for every small mistake I made.’* (S36)
	Extrinsic factors underlying the experience of the critical event	Being in situations of emergency and/or neonatal death/stillbirth	*‘… the woman was in a delirious state: she was not even Italian, so she used expressions in French, and I couldn’t understand her … My tutor sensed something was wrong and checked her blood pressure, which was extremely low; after that, she removed her underwear and she was covered in blood.’* (S33)
		Poor role models	*‘ “Shut up, don’t scream! Don’t move, I cannot place the non-stress test. Can’t you see that you are fully dilated?! You don’t need the epidural, push, and the baby comes out.” … I thought she represented everything that a midwife should not be.’* (S13)
	The effect of tutorial models	Dysfunctional clinical tutoring	*‘Despite my full participation in every labor, she never gave me the opportunity to perform an obstetric examination, despite my frequent requests. My only chance was marked by a reprimand for an initial assessment error, treated as proof of my incompetence.’* (S36)
		No discussion about the event	*‘… everyone just returned to their tasks; there was no discussion or words. For days, I tried to find an explanation for what I had experienced … Nothing seemed to have happened. No words, no “Was it your first time?” no “How do you feel?”. The silence overwhelmed me.’* (S14)
		Functional clinical tutoring	*‘Having found tutors, in subsequent internships, who supported me through my difficulties, along with the feedback from the women themselves, allowed me to leave the negative experiences and continue my studies.’* (S19)
**Theme 2: Suffer the consequences of the event**	Emotional challenges	Negative emotions	*‘Terror, fear, guilt and, at the same time, extreme disbelief. A sense of oppression and feeling powerless to remedy the situation.’* (S09)
		Excessive emotional involvement	*‘I empathized too much with the mother, to the point of having to leave the delivery room because I was crying from how involved I was.’* (S18)
	Late consequences of the event	Difficulty in overcoming the event	*‘The image of that 18-week fetus still comes to mind. Processing that moment took many tears and a lot of time.’* (S14)
		Fear of not being good enough to perform the role	*‘I still don’t know if I will become a midwife that will work in the delivery room, because everything is beautiful as long as everything goes well, but I don’t know if I will be able to handle emergency situations.’* (S15)
		Questioning about continuing the studies	*‘The event made me seriously question my decision, making me believe I had chosen the wrong degree program and career for me.’* (S19)
**Theme 3: Engage in a dynamic process to acquire the professional dimension**	Reasons behind career choice	Interest in the healthcare/midwifery field	*‘I fell in love with this profession and realized it was my path.’* (S01)
		Midwifery as a second choice	*‘Midwifery was not my first choice; I absolutely wanted to get into medicine … but it was the path I felt most aligned with. Today, I know it was the right choice.’* (S02)
	Event processing strategies	Opportunities to discuss the event	*‘However, I got through it thanks to my clinical preceptor, with whom I openly discussed what I had experienced and who advised me on strategies to manage anxiety during labor/delivery.’* (S11)
		External support received	*‘The elements that helped me are my friends and family. Even the women themselves when told me how much I helped them and that I was doing a good job.’* (S23)
		Seeking support beyond the professional sphere	*‘In the following days, I did not receive support despite obvious difficulties and my emotions were only seen as flaws. I asked for help from the person I was working with, but they told me that I had to find a way to overcome my emotions and become more impassive … I underwent psychotherapy for eight months, perhaps that helped me.’* (S18)
	Development of professional identity	Awareness of the event as part of the profession	*‘… aware that by choosing this job, I must consider that there are not only positive moments.’* (S04)
		Strong professional motivation	*‘I decided to complete my studies because becoming a midwife has always been my dream.’* (S15)
		Positive emotions generated by professional practice	*‘Love for this work, the joy I feel witnessing the birth of a new life, the pride I feel when I perform well in certain procedures, and when I receive thanks from the women after they give birth.’* (S06)

S: student.

### Discovering the elements that shape the perception of the event

This theme examines how intrinsic and extrinsic factors, along with the tutorial model, influence midwifery students’ experiences of traumatic events during their clinical training. Intrinsic factors underlying the experience of the traumatic event include a limited understanding of the unfolding situation, where students express confusion and agitation in high-pressure contexts:

*‘I did not understand what was happening, I was very scared.’* (S01)

Alongside a recognition of their lack of experience, they came to terms with the time and practice required to develop composure during emergencies. Students also reveal an acute awareness of their own limitations, acknowledging the challenges of being reprimanded without constructive feedback and the struggle to bridge the gap between theoretical knowledge and clinical practice:

*‘It was my first internship experience in the delivery room, so I didn’t know how to navigate, and there were many things I didn’t know.’* (S19)

Extrinsic factors underline the impact of external pressures on these experiences. Situations involving emergencies or neonatal death often compound the difficulties, as students face communication problems and procedural barriers that increase their stress. The theme also highlights the pivotal role of tutorial models in shaping these perceptions. Dysfunctional clinical tutoring is marked by a lack of opportunities to actively participate in obstetric tasks:

*‘She [the tutor] slapped my hands when I tried to act, and when I did not (out of fear of being intrusive), she berated me in front of the woman and the other midwives.’* (S22)

In addition, there is an absence of meaningful interactions or feedback, leaving students feeling isolated and overwhelmed. On the contrary, examples of functional clinical tutoring underscore how supportive and understanding mentors can foster resilience and encourage students to process and learn from their experiences:

*‘Having found tutors, in subsequent internships, who supported me through my difficulties, along with the feedback from the women themselves, allowed me to leave the negative experiences and continue my studies.’* (S19)

### Suffer the consequences of the event

This theme delves into the emotional and psychological toll of traumatic events on students during their internships. Emotional challenges emerge strongly, with students recounting intense negative emotions such as anxiety, fear, guilt, and powerlessness, alongside instances of excessive emotional involvement, where deep empathy for the patients leads to overwhelming personal distress. One student described this impact, stating:

*‘This event has made me always feel anxious about the delivery room.’* (S20)

The late consequences of these events are profound, as many struggle to process the experiences long after they occur, describing vivid memories that resurface unexpectedly and hinder emotional recovery:

*‘The event still affects me. Even after two and a half years.’* (S16)

These challenges often result in self-doubt, with students fearing they may lack the capability to meet the demands of the midwifery profession and questioning their decision to continue their studies:

*‘… the event made me think, “You will never be able to handle something like that”.’* (S33)

### Engage in a dynamic process to acquire the professional dimension

The third theme captures the proactive efforts students undertake to navigate challenges and shape their professional identity. The reasons behind their career choice reveal a deep connection to the profession, with many expressing a passionate interest in the field and, for some, the realization that midwifery, even when not their first choice, aligns perfectly with their values and aspirations. Event processing strategies play a critical role in helping students manage these experiences, highlighting the importance of open discussions with clinical preceptors and the invaluable support received from family, friends, and even patients, whose gratitude and positive feedback serve as powerful motivators:

*‘The preceptor gave me a lot of confidence, autonomy and reassured me in my uncertainties…’* (S31)

Students often seek support from external sources to process their emotions and rebuild confidence. These sources primarily include personal connections such as friends and family. As one participant expressed:

*‘The elements that helped me are my friends and family.’* (S23)

The development of professional identity emerges as a central aspect of this theme, as students come to recognize that challenging events are an inherent part of the profession:

*‘…understanding I could not do otherwise and accepting that these events are normal in our work’* (S09)

Despite the difficulties, they demonstrate a strong motivation to pursue midwifery, finding inspiration in the positive emotions generated by their practice, such as the joy of witnessing new life, pride in mastering clinical skills, and the fulfilment of making a difference in the lives of others.

## DISCUSSION

This study aimed to explore how the experience of traumatic events influences students’ motivation to continue studying, and the professional expectations associated with the midwife’s role. Our findings resulted in three main themes: ‘Discovering the elements that shape the perception of the event’, ‘Suffer the consequences of the event’, and ‘Engage in a dynamic process to acquire the professional dimension’. These themes share common elements and appear to be deeply interconnected.

The first theme, ‘Discovering the elements that shape the perception of the event’, reveals that students could identify the factors that made the experience traumatic. Extrinsic factors, such as living emergencies, witnessing neonatal deaths or stillbirths, or observing unprofessional behaviors and inadequate patient care, identified feelings of inadequacy and conflicted with students’ expectations of supportive mentorship. These elements have been widely documented in previous studies as traumatic in shaping how events are perceived^10,15^. In the study of Davies and Coldridge^15^, a group of midwifery students from a University in UK described a ‘culture of silence’ as normal in their internship setting, where debriefing after distressing events was uncommon, leaving little time for reflection and learning. Similarly, in our study, the student midwives reported a lack of opportunities to share and reflect on their experiences. Often, no one gave them the chance to discuss their feelings, emotions or, more easily, how they perceived the situation and what they learned from it, even when they actively sought it. Beyond these aspects, other elements directly related to the students, such as lack of experience and poor understanding of what is happening, contributed to how students perceive the event. Students frequently find themselves overwhelmed by situations they are not yet equipped to handle, leading to confusion and disorientation. This combination of factors – the lack of emotional support and opportunities for reflection, together with inexperience and poor understanding of situations – can intensify students’ feelings of helplessness and vulnerability, making the experience not only challenging but also potentially traumatic.

In the abovementioned study^15^, midwifery students also reported feeling powerless when witnessing poor or disrespectful care for women. Our study echoes these findings, as students highlighted negative professional examples in their narratives. However, in our case, the students not only disagreed with these behaviors but firmly stated that these professionals did not embody what a midwife should be, expressing the desire to change their environment, fearing that they might become like them. It is precisely these negative professional examples that lead students to gradually distance themselves from their initial expectations regarding the professional role. Witnessing behaviors that contradict their ideals creates a sense of disillusionment. Over time, this discrepancy between expectations and reality may result in frustration, disengagement, or even a shift in their professional aspirations, leading them to consider leaving the study program. This aligns with the concept of burnout, which Edú-Valsania et al.^23^ described as an occupational phenomenon resulting from chronic stress in the workplace, potentially leading to disengagement and changes in career intentions.

Although various factors influence students’ perception of traumatic events, the role of the clinical preceptor remains particularly significant^24^. Whether the tutoring model is functional or dysfunctional, it has the potential to profoundly shape the students’ experience of the event and influence their learning process. It is widely recognized that negative professional attitudes, as well as workplace bullying, could impact the students’ experiences in their clinical settings: mentors’ negative behavior often made students feel belittled, like a burden, unsupported, and unsupervised, suggesting that negative clinical mentorship experiences can hider learning and lead to pedagogical breakdown^10,12^. Many students mentioned feeling uncomfortable when closely examined and embarrassed in front of other midwives or patients because they had not yet mastered certain theoretical or clinical competencies. Similarly, a dysfunctional tutoring model can negatively impact the learning process, while a positive relationship between the mentor and the student has been documented to have a beneficial influence: a good relationship between the student and mentor has been found to be protective against student dropout, fostering development of resilience in the student. Mentors serve as role models, exemplifying the values and interpersonal skills that students need to demonstrate in their clinical practice^25^. Students express a sense of gratitude toward midwifery role models who provided support and guidance during challenging moments of their internship. These mentors are frequently recognized for encouraging and motivating students to persevere in their educational journey. While the role of the clinical preceptor clearly emerges as central in students’ experiences, it is important to recognize that student retention in midwifery is influenced by multiple, interconnected factors. According to Dawson et al.^26^, woman-centered philosophy, strong professional identity, career expectations, passion for the role and emotional fulfilment contribute to shaping students’ intentions to remain in the profession. Furthermore, the Global Strategic Directions for Nursing and Midwifery report underscores the importance of integrated strategies to strengthen midwifery retention. These strategies include promoting psychological wellbeing, fostering professional development, improving education, and ensuring supportive organizational conditions^27^.

The second theme, ‘Suffer the consequences of the event’, highlights the event’s impact on students, both in terms of the emotional challenges they had to face and the uncertainty it caused about continuing their studies. Students have strong emotional responses to traumatic events and struggle to manage their feelings^24^. In all their descriptions, students express a range of negative emotions, including anxiety, fear, agitation, anger, frustration and guilt. These feelings may arise from the event itself or their interactions (or lack of interactions) with their mentor. They also report becoming overly emotionally involved and finding it difficult to move past the event, which, in some cases, continues to affect them. The impact of these experiences is so significant that it leads them to question their career choice and doubt their ability to become midwives in the future. As previously mentioned, these feelings often stem from a lack of tools and knowledge to understand and cope with the dynamics of the event.

The third theme, ‘Engage in a dynamic process to acquire the professional dimension’, addresses the dynamic process of acquiring a professional dimension, going through a moment of processing the event and developing more awareness of the professional role. In line with previous research findings^24^, the students described their strategies for processing the event through discussion and reflection with their tutor, as well as by seeking support from those around them and turning to external help outside the professional domain. Some students reported seeking assistance from mental health professionals to cope with the aftermath of the event and receive ongoing support. One of the outcomes of processing the event, particularly in cases involving emergencies and perinatal deaths, is an increased awareness that such occurrences are an inherent part of the profession. This awareness highlights the need to learn how to cope with these situations. Notably, findings from an integrative review conducted by Oates et al.^24^, suggest that exposure to stressful and traumatic situations during midwifery training can also catalyze professional growth. Despite many of them questioning whether to continue their studies after experiencing traumatic events, this theme reveals the passion these students feel for the profession. Students show a strong desire to continue, driven by the satisfaction they gain from the women they care for and the positive emotions they experience in their work. This passion, along with the value midwives place on forming a bond with the women they support, has been documented as the primary source of fulfilment for those entering the profession, many of whom view midwifery as a true calling^28^.

### Limitations

The study has several limitations. An open-ended questionnaire was chosen as the data collection method because it was simpler to administer compared to interviews, which would have required more time and resources. This approach limited the ability to further explore the students’ narratives and precluded the observation of non-verbal communication. Furthermore, the participants were drawn from different clinical internship settings. Two of the hospitals where the students attended their training were relatively small, with an annual birth rate of 500 to 1000, resulting in a lower volume of obstetric pathology cases and a reduced workload. This may have impacted the nature of the experiences reported by the students. Another limitation is the lack of exploration of clinical preceptors’ perspectives, which would be valuable in understanding their views on the student’s experiences of traumatic events. This last aspect is undoubtedly a starting point for future research.

## CONCLUSIONS

Students who experience traumatic events during their internships may, in certain cases, question whether to continue their studies, particularly when confronted with dysfunctional tutoring or unprofessional role models. Nevertheless, their strong motivation and passion for the profession – nurtured by the satisfaction and positive feelings arising from providing care to women – often lead them to persevere and realize their aspiration of becoming midwives.

This study offers a comprehensive overview of this phenomenon within a university in northern Italy. However, several of the results align with those from studies conducted in the UK and internationally, indicating potential commonalities on this issue across various countries. Examining how students perceive traumatic events and subsequent responses is particularly important for clinical preceptors. This insight can help tutors provide more effective guidance and support to students, before they consider leaving the profession, thereby contributing to the increasingly prevalent global issue of intent to leave.

## Supplementary Material



## Data Availability

The data supporting this research are available from the authors on reasonable request.
