# Supplementary material for: How traumatic internship experiences shape midwifery students' motivation and career expectations: Findings from a qualitative study
Source: Eur J Midwifery. 2025 Jul 3;9:10.18332/ejm/205672. doi: 10.18332/ejm/205672 (PMC12224113; doi:10.18332/ejm/205672)
Supplement: Supplementary file 1 [file EJM-9-32-s1.pdf]

Supplementary Table 1
Open-ended Questionnaire.

Socio-demographic information about participants:<br><br>- Age<br>- Gender (Male, Female)<br>- The student exceeds the standard time to graduate (Yes, No)
1. Please, briefly explain the main reason why you enrolled in the Midwifery bachelor's Degree Programme.
In the following sections, I ask you to recall an event that, if occurred during your internship, had an emotional/personal impact on you and consequences that led you to question your motivation and expectations regarding the role of the midwife and the bachelor's degree program you attending…
2. What happened? Please, briefly describe what occurred to clarify the nature of the event.
3. Please, describe the subjects present in the context and the behaviors that contributed to making the experience of the event critical for you.
4. Please, describe how you felt and the emotions you experienced at the time of the event.
5. What consequences did the event have on you? For how long?
6. What strategies did you adopt to cope with the consequences the event had on you? You can describe which factors influenced your decision to continue your studies.

© 2025 Dato M. et al.
